# Low LPA gene kringle IV-2 repeat copy number association with elevated lipoprotein (a) concentration as an independent risk factor of coronary atherosclerotic heart disease in the Chinese Han population

**DOI:** 10.1186/s12944-018-0753-1

**Published:** 2018-05-10

**Authors:** Lishan Sun, Ming Zong, Cuncun Chen, Lihong Xie, Fei Wu, Ming Yu, Lieying Fan

**Affiliations:** Department of Clinical Laboratory, Shanghai East Hospital, Tongji University School of Medicine, No. 150, Jimo Road, Shanghai, 200120 People’s Republic of China

**Keywords:** Lipoprotein(a), Atherosclerotic heart disease, Kringle IV type 2 repeat

## Abstract

**Background:**

Lipoprotein (a) [Lp(a)], which is genetically determined by the LPA gene kringle IV type 2 (KIV-2) repeat copy number, has previously been reported in different populations. However, it is uncertain if the same occurs in the Chinese Han population. This study explored the correlation of Lp(a) mass or particle concentration with KIV-2 repeat copy number and application for coronary atherosclerotic heart disease (CAHD) risk assessment.

**Methods:**

A cross-sectional study including 884 subjects was conducted. The Lp(a) level and routine risk factors of CAHD were compared. The KIV-2 copy number distribution, relationship with Lp(a), and assessment for CAHD risk were explored.

**Results:**

The mean of Lp(a) mass or particle concentration in the CAHD group was higher than that in the non-CAHD group, while the KIV-2 copy number in the CAHD group was lower. Lp(a) had auxiliary values in gauging the type of plaque and was significantly higher in the soft-plaque group than that in the other two groups (200 mg/L [21.5 nmol/L], 166 mg/L [18.6 nmol/L], 149 mg/L [17.1 nmol/L], respectively, *P* < 0.05). Kappa test indicated divergence for the same individual using two Lp(a) concentrations (kappa value was 0.536 [< 0.75]). Elevated Lp(a) was an independent CAHD risk factor, whatever mass or particle concentration, and large KIV-2 copy number was a protective factor.

**Conclusion:**

Lp(a) level and small KIV-2 copy number are risk factors for CAHD in the Chinese Han population; furthermore, elevated Lp(a) may gauge the type of coronary plaque.

## Background

Several studies have confirmed that there is a close relationship between high lipoprotein (a) [Lp(a)] level and coronary atherosclerotic heart disease (CAHD) [1, 2]. These studies have also provided three levels of evidence. In the first level, a large number of case-control studies, cohort studies and large-sample-size epidemiological surveys as well as clinical meta-analyses have confirmed that there is a close relationship between individual Lp(a) level elevation and CAHD risk, cardiovascular event probability elevation and poor prognosis [3–6]. Evidence at the second level is based on large-sample-size pan-genomics studies, which correlate “single nucleotide polymorphism (SNP) and repeat copy number variation (CNV) in LPA gene” with “Lp(a) levels and CAHD occurrence.” They These studies also provide direct genetic evidence about the relationship between high Lp(a) level and CAHD [7–9]. At the third level, evidence is based on the results of Mendelian randomized studies confirming that Lp(a) levels have a genetic predisposition in a given population, i.e., most of the Lp(a) determinant levels result from LPA gene mutations [10–12]. However, since a therapeutic regimen for effectively lowering Lp(a) is not currently available, direct evidence of lowering CAHD risk by lowering Lp(a) levels has not yet been determined detected in clinical practice. Whether patients with high Lp(a) need to receive treatment for lowering Lp(a) is still controversial, but many therapeutic clinical trials are underway [13–15]. The molecular structure of Lp(a) is similar to that of LDL and is composed of specific apo (a) molecules and LDL-like particles attached via a disulfide bond. The physiological functions of Lp(a) are unclear [16], and whether Lp(a) is an independent risk factor for CAHD has not been fully elucidated. There are two expression pathways for detecting Lp(a)-related results: mass concentration and particle concentration (briefly, particle concentration). This issue further increases clinical study complexity with uncertain results [17]. Additionally, large-sample-size studies were recently conducted for exploring the clinical value of particle concentration but were inconclusive [18]. There is scant study on how LPA gene’s kringle IV-2 (KIV-2) repeat copy number variation is distributed and affects Lp(a) concentration along with CAHD risk factor in the Chinese population [19, 20]. This study compared the difference between CAHD risk assessment by using Lp(a) mass concentration and particle concentration, and then established a method to measure the KIV-2 repeat copy number and explore the distribution in the Chinese Han population, the relationship with lp(a) concentration as well as risk assessment for CAHD.

## Methods

### Study subjects

The subjects were selected from patients who visited the Department of Cardiology in Shanghai East Hospital from October 2013 to January 2017. These patients had undergone coronary arterial computerized tomography angiography (CTA) for suspected coronary artery disease. Patients receiving nicotinic acid were excluded from this study. All 884 patients enrolled were of Chinese Han ethnicity including 456 males (51.6%) and 428 females (48.4%), with an average age of 59 ± 9 years. The experimental protocol was reviewed and approved by the Hospital Ethics Committee, and all enrolled subjects signed informed consents.

The coronary arterial CTA examination results were independently gauged and determined by two deputy chief physicians or similar level staff members. Clinical data of patients were then recorded, including gender, age, coronary arterial CTA examination result, etc. All subjects were divided into two CAHD and non-CAHD groups depending on coronary arterial CTA results and whether coronary atherosclerotic plaque was detected. Furthermore, based on the CT value (Hounsfield unit, HU) of coronary atherosclerotic plaque in the stenotic position, the patients in the CAHD group were divided into 3 subgroups: soft plaque (< 60 HU), calcified plaque (> 130 HU) and mixed plaque (60~ 130 HU). The criteria for dividing subgroups were as follows. For patients who had coronary atherosclerotic plaque in various coronary artery branches and trunks, a patient with soft plaque in one or more locations was included in the soft-plaque subgroup; a patient with calcified plaque in one or more locations was included in the calcified-plaque subgroup; a patient with no soft plaque or with only a mixed plaque (or more), or with both a mixed plaque (or more) and a calcified plaque (or more), was included into the mixed-plaque subgroup.

### Conventional clinical and laboratory indicator tests

Subjects had to fast for more than 8 h before coronary arterial CTA examination. Venous blood was collected, and the samples were centrifuged at 1880×g for 10 min. Sera were separated for conventional biochemical tests, including Lp(a) particle concentration, Lp(a) mass concentration, triglycerides, total cholesterol, high density lipoprotein cholesterol (HDL-C), low density lipoprotein-cholesterol (LDL-C), and apolipoprotein B (Apo(B)); hypersensitive C-reactive protein (hs-CRP) and non-HDL-C were calculated by HLD-C subtracted from total cholesterol. Further, EDTA anticoagulated whole blood was collected from patients for HBA1c tests and individual genomic DNA extraction. Lp(a) concentrations were measured in serum using an immunoturbidimetric method. Interassay variation for samples in the centralized laboratory was < 7%. The reference material is SRM2B IFCC/WHO for the two Lp(a) assay reagents. All results were measured on the condition of quality control being normal.

KIV-2 repeat copy number was determined according to the repeat copy number, using a Taqman probe-based quantitative PCR technique, as reported in the literature [21]. The basic detection steps are as follows: Specific probes for KIV-2 and endogenous-single-copy control housekeeping gene were synthesized. Applied Biosystems® 9600 Real-Time PCR Systems was used with a total reaction system of 20 μl, which included premix Ex Taq (2×) 10 μl, specific primer 0.4 μl, Taqman probe 0.8 μl, and DNA template 2 μl (concentration 3–5 ng/μl).

### Statistical analysis

Statistical analysis was conducted using SPSS 18.0 software. Data with Gaussian distribution were expressed as the mean ± standard deviation (SD), and data with non-Gaussian distribution were expressed as the median (interquartile range). For further statistical analysis, data with non-Gaussian distribution log transformed into Gaussian distribution. An independent sample t-test or one-way analysis of variance (ANOVA) was used to compare the mean of each group; the *χ*^2^ test in nonparametric statistics was used to compare categorical variables among groups. Logistic regression (backward-stepwise method) analysis was used to estimate CAHD risk for various indicators. This study considered *P* < 0.05 as the significance criterion.

## Results

### Clinical data and characteristics of study subjects

A total of 884 subjects were enrolled in this study, including 484 CAHD patients and 400 non-CAHD individuals. Table 1 shows clinical characteristics and risk factors of all subjects, including gender, age, blood-lipid indicators, hs-CRP, Lp(a) mass concentration and particle concentration as well as KIV-2 copy number. For CAHD and non-CAHD groups, an independent-sample t-test was used to compare the mean of various indicators with Gaussian distribution, and the *χ*^2^ test was used to compare the difference in rates of categorical variables. The means of Apo(B), Lp(a) mass concentration and particle concentration, KIV-2 copy number, HDL-C, age, HBA1c and hs-CRP of patients in the CAHD group were all significantly higher or lower than the non-CAHD group (*P* < 0.05). Differences between the means of the two groups were not significant for other indicators, such as total cholesterol, LDL-C, non-HDL-C and triglycerides. Indicators with differences were believed to be possible risk factors for CAHD. Both average Lp(a) mass concentration and particle concentration of patients in the CAHD group were higher than in the non-CAHD group, while the KIV-2 copy number of patients in the CAHD group was lower than the non-CAHD group. This result fully confirmed that high Lp(a) level and low KIV-2 copy number were closely related to the occurrence of CAHD and were possibly independent risk factors for CAHD.

**Table 1:** Baseline clinical characteristics and risk factors in patients with CAHD and without

	CAHD group *n* = 484 (54.8%)	Non-CAHD group *n* = 400 (45.2%)	t/χ^2^	*P* value
Age(y)	62.46 ± 9.06	56.39 ± 9.57	−9.405^c^	0.000
Sex (male), n (%)	277(57.2%)	170(42.5%)	19.635^b^	0.000
Diabetes mellitus,n(%)	108(22.3%)	28(7.0%)	39.451^b^	0.000
Total cholesterol (mmol/L)	4.79 ± 1.08	4.84 ± 0.98	0.710	0.478
LDL-C (mmol/L)	3.15 ± 0.99	3.15 ± 0.89	0.016	0.988
HDL-C (mmol/L)	1.33 ± 0.59	1.42 ± 0.41	2.661^c^	0.008
non-HDL-C (mmol/L)	3.46 ± 1.17	3.42 ± 0.94	−0.593	0.553
Triglycerides (mmol/L)	1.99 ± 1.27	1.83 ± 1.27	−1.801	0.072
Apo(B) (g/L)	1.09 ± 0.35	1.05 ± 0.31	−1.847	0.065
Lp(a)-mass (mg/L)^a^	181(69–346)	130(66–287)	−3.121^c^	0.002
Lp(a)-particle (nmol/L)^a^	19.1(8.8–44.9)	14.4(7.6–32.3)	−2.543^c^	0.011
KIV-2 copy numbers (copies)	14.11 ± 6.13	15.14 ± 7.11	2.205^c^	0.040
HbA1C (%)	6.12 ± 1.02	5.83 ± 0 .66	−4.824^c^	0.000
hs-CRP (mg/L)^a^	0.91(0.44–2.02)	0.68(0.38–1.62)	−2.967^c^	0.003

Continuous variables are expressed as mean ± SD or ^a^ median (interquartile range) and compared using ‘t’ test

^b^Categorical variables are expressed a numbers (%) and compared using the chi-square test

^c^Compared with control group, P < 0.05

### Comparison of various clinical indicators among subgroups with different plaque types

ANOVA and *χ*^2^ test results for soft-plaque, calcified-plaque and mixed-plaque subgroups were as follows: There were significant differences in the means of respective sub-groups for age, gender, total cholesterol, LDL-C, non-HDL-C, and Lp(a) mass concentration and particle concentration. The results are shown in Table 2. Further intergroup pair-wise comparisons are as follows: Lp(a) levels expressed as mass concentration and particle concentration in the soft-plaque subgroup were higher than the other two groups. Total cholesterol in the soft-plaque subgroup was higher than the mixed- plaque subgroup. Non-HDL-C in the soft-plaque subgroup was higher than in the calcified- plaque subgroup and between-group differences were not significant for the remaining indicators.

**Table 2:** Relationship between risk factors and different nature plaques in CAHD patients compared using ANOVA (n = 484)

	Soft plaque *n* = 259	Calcified plaque *n* = 129	Mixed plaque *n* = 96	*P* value	*P*_1_ value	*P*_2_ value	*P*_3_ value
Age (years)	61.37 ± 9.13	63.41 ± 8.72	64.38 ± 8.88	0.010	0.045	0.006	0.446
Sex (male), n (%)	145(56%)	64(49.6%)	68(70.8%)	0.020^b^	/	/	/
Total cholesterol (mmol/L)	4.90 ± 1.11	4.68 ± 1.01	4.62 ± 1.04	0.045	0.080	0.028	0.640
LDL-cholesterol (mmol/L)	3.27 ± 1.01	3.08 ± 0.88	2.93 ± 0.99	0.009	0.080	0.003	0.266
HDL-cholesterol (mmol/L)	1.32 ± 0.38	1.42 ± 0.67	1.24 ± 0.43	0.076	0.110	0.275	0.026
Non-HDL cholesterol (mmol/L)	3.58 ± 1.11	3.27 ± 1.36	3.37 ± 1.05	0.041	0.017	0.139	0.498
Triglycerides (mmol/L)	2.00 ± 1.26	1.80 ± 1.04	2.20 ± 1.52	0.076	0.168	0.181	0.023
Apo(B) (g/L)	1.12 ± 0.34	1.06 ± 0.38	1.06 ± 0.31	0.178	0.128	0.144	0.983
Lp(a)-mass, (mg/L)^a^	200(69–383)	166(77–293)	149(64–279)	0.025	0.049	0.018	0.662
Lp(a)-particle, (nmol/L)^a^	21.5(5.3–52)	18.6(5.3–43.1)	17.1(7.6–28.01)	0.006	0.038	0.003	0.391
KIV-2 copy numbers (copies)	14.31 ± 6.94	13.84 ± 5.25	13.87 ± 4.20	0.768	0.543	0.587	0.979
HbA1C (%)	6.10 ± 1.08	6.12 ± 0.89	6.18 ± 0.99	0.783	0.848	0.485	0.657
HS-CRP (mg/L)^a^	0.91(0.45–1.79)	0.86(0.43–1.88)	0.92(0.45–2.52)	0.515	0.292	0.456	0.814

Notice: *P* value calculated among three groups; *P*_1_ value compared between soft and calcified plaque; *P*_2_ value compared between soft and mixed plaque; *P*_3_ value compared between calcified and mixed plaque

^a^Results shown as mean ± SD or median (interquartile range)

^b^Results compared using the chi-square test

### Linear regression results of Lp(a) mass concentration and particle concentration

**Fig. 1:**
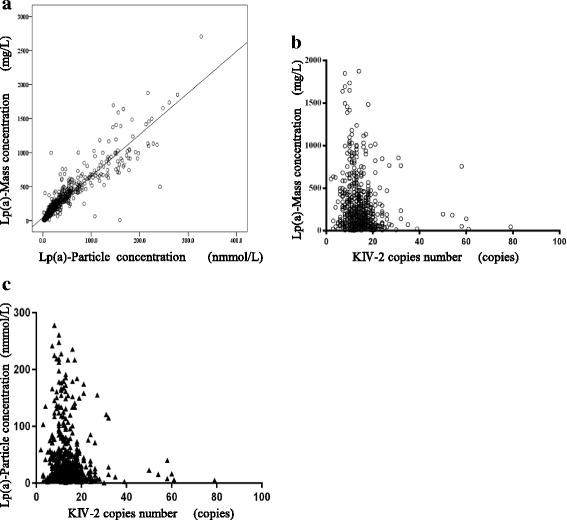
**a** Shows an XY scatter plot of Lp(a) mass and particle concentration, and the linear relationship with a regression equation: Y_Lp(a)-mass_ = 6.565X_Lp(a)-particle_, R^2^ = 0.852, *P* < 0.001. **b** Shows an XY scatter plot of KIV-2 copy number and Lp(a) mass concentration for all participants. The abscissa is KIV-2 copy number, and the left ordinate is Lp(a) mass concentration. **c** Shows KIV-2 copy number and Lp(a) particle concentration. The abscissa is KIV-2 copy number, and the left ordinate (right axis) is Lp(a) particle concentration. There is a negative correlation between Lp(a) level and KIV-2 copy number, i.e., the smaller the KIV-2 copy number the higher the Lp(a) concentration

For correlation analysis of all subjects and to draw a scatter plot, Lp(a) particle concentration was considered the abscissa and Lp(a) mass concentration was considered the ordinate for linear regression analysis, and results are indicated in Fig. 1a. The results of two different concentrations showed a good linear relationship, and the linear regression equation of particle concentration to mass concentration could be obtained: Y_Lp(a)-mass_ = 6.565X_Lp(a)-particle_, and the correlation coefficient R^2^ was 0.852 (*P* < 0.001). At present, abnormal Lp(a) levels in clinical practice are mainly evaluated based on a reagent instruction for use. For Lp(a) mass concentration, 300 mg/L is usually taken as a cutoff value, i.e., an individual’s test result ≤300 mg/L is considered normal and > 300 mg/L is considered elevated. For particle concentration, 75 nmol/L is the cutoff value, i.e., ≤75 nmol/L is normal, and > 75 nmol/L is considered elevated. According to the criteria, all subjects were gauged as normal or abnormal in Lp(a) mass concentration and particle concentration, respectively. The number and proportion of normal and abnormal cases were respectively counted. Kappa consistency test was then used to compare the consistency between the two kinds of Lp(a) results. The number of subjects with elevated Lp(a) mass concentration was significantly higher than that of subjects with elevated particle concentration (231 vs. 108 in 884 subjects), and the calculated kappa value from the consistency test was 0.536 (*P* < 0.01). Considering kappa value > 0.75 the acceptable criterion for consistency, judgment of the same patient on the basis of two Lp(a) concentrations was obviously inconsistent. Therefore, varying judgments on the basis of two concentrations bring complexity to the clinician’s decision in assessing risk and evaluating the effect of treatment in CAHD patients.

### The relationship between Lp(a) concentration and KIV-2 copy number distribution in the Chinese Han population

Considering the LPA gene KIV-2 copy number the abscissa and Lp(a) particle concentration and mass concentration the ordinates, an XY scatter plot for all subjects was drawn, as shown in Fig. 1b . Pearson correlation analysis showed that the coefficient r between particle concentration or mass concentration and KIV-2 copy number was − 0.145 or − 0.135, respectively. Both variables had *P* < 0.01, indicating that the Lp(a) level was negatively related to the KIV-2 copy number: The larger the KIV-2 copy number was, the lower the Lp(a) concentration. This conclusion was accurate for the Lp(a) levels described based on both particle concentration and mass concentration. Numerically, particle concentration was more closely related to KIV-2 copy number than mass concentration.

**Fig. 2:**
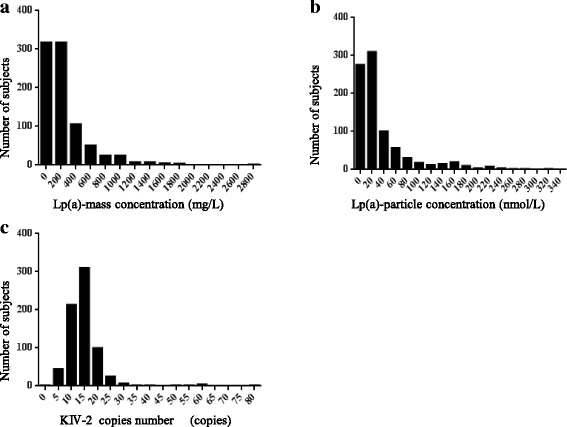
Represents Lp(a) mass concentration and particle concentration, and KIV-2 population distributions in the Chinese Han ethnic population; **a** Lp(a) mass concentration distribution histogram; **b** Lp(a) particle concentration distribution histogram; **c** KIV-2 copy number distribution histogram

Distribution histograms of Lp(a) mass concentration and Lp(a) particle concentration as well as KIV-2 copy number were drawn via software in all study subjects, as shown in Fig. 2. For all subjects, median Lp(a) mass concentration was 146 mg/L (interquartile range: 67–342 mg/L), median Lp(a) particle concentration was 17 nmol/L (interquartile range: 8–41 nmol/L), and median KIV-2 copy number was 14 (interquartile range: 10–17 copies), respectively.

### Differences in average Lp(a) concentration and CAHD patient proportion among groups with different KIV-2 copy numbers

**Fig. 3:**
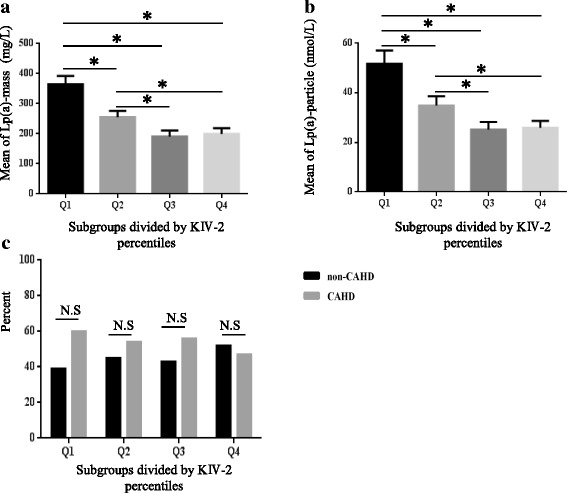
Comparison of Lp(a) mass concentration, particle concentration, and percent of CAHD among KIV-2 subgroups; * *P* > 0.05; N.S: no significance; **a** Differences of Lp(a) mass concentration in CAHD and non-CAHD participants among different KIV-2 subgroups; **b** Differences of Lp(a) particle concentration in CAHD, non-CAHD participants among different KIV-2 subgroups; **c** Differences of proportion in CAHD, non-CAHD participants among different KIV-2 subgroups

All subjects were divided into four groups: Groups Q1-Q4 were based on interquartile range of the KIV-2 copy number (measured in the laboratory); thus, the 25th, 50th and 75th percentiles were 10, 14, and 17 copies, respectively. ANOVA results indicated that the among-group mean Lp(a) was significantly different, except between the last two groups, and the mean in group Q1 was the highest. In the comparison of CAHD prevalence among different groups, the *χ*^2^ test results showed there was no significant difference in CAHD percent among the groups (Pearson Chi-square value was 5.710, *P* = 0.127). All results are shown in Fig. 3.

### Risk estimation of various clinical conventional risk factor indicators and Lp(a) and KIV-2 copy number on CAHD occurrence

**Fig. 4:**
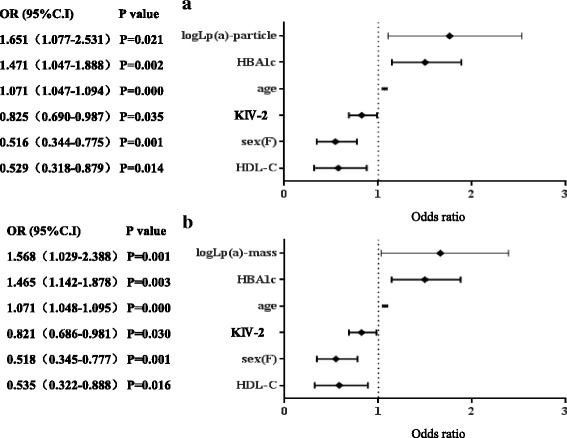
Tree diagrams of logistic regression analysis on various possible risk factors for CAHD; **a** Analysis results of Lp(a)-particle and other indicators; **b** Analysis results of Lp(a)-mass and other indicators

For all subjects, dependent or independent variables were explored for CAHD, considering all CAHD risk factors, such as gender, age, KIV-2 copy number, HDL-C, Lp(a) mass concentration/particle concentration, conventional lipid indicators, and hsCRP and HBA1c as independent variables. The backward-stepwise method was used for logistic regression analysis to calculate respective odds ratios (OR). In the final regression equation, 6 indicators, KIV-2 copy number, Lp(a), HBA1c, age, gender and HDL-C, were retained. High Lp(a) levels (both mass concentration and particle concentration), high HBA1c and increasing age were independent risk factors for CAHD. In contrast, high HDL-C, large KIV-2 copy number and female gender were protective factors for CAHD. A tree diagram of the statistical results is shown in Fig. 4. The OR value calculated by using particle concentration was greater than the OR value calculated by using mass concentration, indicating that Lp(a) levels expressed by particle concentration were more closely related to CAHD than mass concentration.

## Discussion

Since its discovery, elevated Lp(a) seems to be related to CAHD but remains controversial, as Lp(a) is an independent risk factor for CAHD due to its structural peculiarity and complexity [22, 23]. Some studies even consider that Lp(a) is related to the severity of CAHD [4, 24, 25]. This study indicates that in addition to age, gender, HBA1c, hs-CRP and other conventional risk factors, the difference in Lp(a) levels between CAHD and non-CAHD groups was significant, as both particle concentration and mass concentration in the CAHD group were significantly higher than in the non-CAHD group. This result indicates that in the Chinese Han population, elevated Lp(a) is also an independent risk factor for CAHD and is consistent with the conclusion of a cross-sectional study on the Chinese Han population by Cai et al. [26]. For comparison, the results obtained from various subgroups with different plaque types indicated significant differences in age, gender, cholesterol, LDL-C, non-HDL-C and Lp(a) levels in the soft-plaque subgroup, especially Lp(a) particle concentration, which had particular value in predicting soft plaque. This outcome was a new discovery in the clinical application of Lp(a). Compared to coronary angiography, the CTA examination used in this study could determine the type of atherosclerotic plaque and assess its stability in addition to being convenient and causing very little damage. An unstable plaque is prone to rupture, thus promoting thrombus formation and causing a serious cardiovascular event. Hence, identifying coronary arterial soft plaque helps identify unstable plaque. Hopefully, the correlation between Lp(a) levels and soft plaque makes Lp(a) an auxiliary indicator for gauging the stability of atherosclerotic plaque.

This study found good linear relationship between two Lp(a) concentrations by comparing the two and deduced that the conversion equation was: mass concentration = particle concentration × 6.565. However, the recommended conversion factor in the reagent instruction for use was 4.167, which was significantly different from the estimated conversion factor. Additionally, while using two concentrations of Lp(a) to gauge the same individual based on their respective reference intervals, i.e., mass concentration ≤ 300 mg/L and particle concentration ≤ 75 nmol/L, the conclusions were not exactly the same. The proportion of patients with elevated mass concentration was significantly higher than the proportion of patients with elevated particle concentration, indicating that mass concentration may overestimate the Lp(a) level of the patient, or that the biological reference interval with particle concentration ≤ 75 nmol/L is not suitable for the Chinese Han population. Therefore, interchangeability between particle concentration and mass concentration needs to be studied further in a large sample size to establish a biological reference interval suitable for the Chinese Han population. Currently, direct conversion is not suitable, and particle concentration should be used to report results as far as possible. Some studies even propose that total mass concentration of Lp(a), as an indicator, should not be advocated [27].

The present study indicates that the KIV-2 copy number variation of the LPA gene is the key factor determining Lp(a) concentration, and its copy number varies from 2 to > 40 in the Chinese population. Some studies have indicated that there is racial heterogeneity in LPA gene SNP loci [28, 29]. Therefore, it is hypothesized that there may be racial differences in the KIV-2 copy number. In this study, Lp(a) levels and KIV-2 copy number distributions in the Chinese population were observed. The results showed that the median Lp(a) mass concentration was 146 mg/L and that of particle concentration was 17 nmol/L in the Chinese population, which was significantly lower than those in other racial populations from different countries, prompting that Lp(a) distribution in the Chinese population has particularity. The Lp(a) biological reference interval is established based on the 75th percentile of Caucasians. KIV-2 copy number distribution in the Chinese Han population with an average copy number of 14 is also different from other races [30]. As KIV contains 9 other single-copy repeats, in addition to KIV-2, the KIV sequence actually contains 23 repeats. The present study also confirmed the negative relationship between KIV-2 copy number and Lp(a) concentration. Linear regression analysis showed that the larger the KIV-2 copy number, the lower the Lp(a) concentration in an individual’s blood, which is consistent with Paultre et al.’s study conclusion that apo (a) subtype with fewer KIV copy numbers is related to higher Lp(a) concentration and is a risk factor, thus predicting CAHD [31]. The population was then divided into four groups based on KIV-2 copy number and quartile: The results showed that mean Lp(a) concentration in groups Q1 and Q2 with fewer copy numbers was higher than that in groups Q3 and Q4. Additionally, this number was higher in group Q1 than in group Q2, and there was no significant difference between groups Q3 and Q4, indicating continuous change between KIV-2 copy number and Lp(a) concentration, with a cumulative effect.

Considering the CAHD risk factors as dependent or independent variables, logistic regression was performed, and the results were as follows. Besides high HBA1c,advanced age, low HDL-C level, male gender and other factors, such as high Lp(a) level and low KIV-2 copy number, were also risk factors for CAHD occurrence. Elevated Lp(a) level had the highest OR value for CAHD and large KIV-2 copy number could lower the risk. Additionally, analysis of CAHD patients in groups with different KIV-2 copy numbers revealed no significant difference among the different groups in this study, indicating that the KIV-2 copy number had no significant effect on CAHD. This finding was inconsistent with the logistic regression results, which indicated the relationship between the KIV-2 copy number at the gene level and CAHD. Effects of the KIV-2 copy number at the gene level, however, are limited. KIV-2 copy number variation also needs other factors such as epigenetic, protein expression, assembly and environment to exert effects collectively. Additionally, there are also reports about uncertain causality between LPA gene mutation and CAHD [8, 32].

Since this study included cases and controls, biases may have been introduced. The accuracy of KIV-2 copy number detection was also one of the factors affecting the analysis of results. This study used the qPCR technique developed by Lanktree et al. [21, 33], however, it measured the average KIV-2 copy number of an individual diploid. Since two haploids might contain different KIV-2 copy numbers, this method cannot effectively differentiate between the effects of individual haploids [34]; for example, the 16/28 genotype had the same PCR measurement result as the 22/22 genotype, but gene expression or patients’ phenotype might be different between the two gene types.

## Conclusions

In summary, the present study explored the relationship between “Lp(a) particle concentration and mass concentration distribution” and “CAHD” in the “Chinese Han population”, confirming that high Lp(a) concentration is a risk factor for CAHD and that Lp(a) particle concentration has auxiliary diagnostic value to gauge the type of coronary arterial plaque. A qPCR technique was used to detect the KIV-2 copy number of subjects and its relationship with Lp(a) concentration, and its population distribution was then assessed as well as its role in risk assessment of CAHD.

## Abbreviations

CAHD: Coronary atherosclerotic heart disease
CNV: Copy number variation
CTA: Computerized tomography angiography
HDL-C: High density lipoprotein-cholesterol
hs-CRP: Hypersensitive C reactive protein
HU: Hounsfield unit
KIV-2: Kringle IV type 2
LDL-C: Low density lipoprotein-cholesterol
Lp(a): Lipoprotein(a)
SNP: Single nucleotide polymorphism
